# Succeeding in New Vaccine Introduction: Lessons Learned From the Introduction of Inactivated Poliovirus Vaccine in Cameroon, Kenya, and Nigeria

**DOI:** 10.1093/infdis/jiw544

**Published:** 2017-06-30

**Authors:** Soleine Scotney, Sarah Snidal, Yauba Saidu, Abiola Ojumu, Antony Ngatia, Murtala Bagana, Faith Mutuku, Joelle Sobngwi, Oniovo Efe-Aluta, Julia Roper, Yann LeTallec, Alice Kang’ethe

**Affiliations:** 1 Vaccines Delivery Team, Clinton Health Access Initiative, Nairobi, Kenya;; 2 Vaccines Delivery Team, Clinton Health Access Initiative, Kampala, Uganda;; 3 Vaccines Delivery Team, Clinton Health Access Initiative, Yaounde, Cameroon;; 4 Vaccines Delivery Team, Clinton Health Access Initiative, Abuja, Nigeria; and; 5 Vaccines Delivery Team, Clinton Health Access Initiative, Zurich, Switzerland

**Keywords:** Polio, IPV, NVI, technical working groups, RI, program management.

## Abstract

Introducing a new vaccine is a large-scale endeavor that can face many challenges, resulting in introduction delays and inefficiencies. The development of national task teams and tools, such as prelaunch trackers, for the introduction of new vaccines (hereafter, “new vaccine introductions” [NVIs]) can help countries implement robust project management systems, front-load critical preparatory activities, and ensure continuous communication around vaccine supply and financing. In addition, implementing postlaunch assessments to take rapid corrective action accelerates the uptake of the new vaccines. NVIs can provide an opportunity to strengthen routine immunization, through strengthening program management systems or by reinforcing local immunization managers’ abilities, among others. This article highlights key lessons learned during the introduction of inactivated poliovirus vaccine in 3 countries that would make future NVIs more successful. The article concludes by considering how the Immunization Systems Management Group of the Global Polio Eradication Initiative has been useful to the NVI process and how such global structures could be further enhanced.

In May 2012, the World Health Assembly declared the eradication of poliovirus to be a programmatic emergency for global public health [1]. This eradication requires a globally coordinated effort to shift from the use of oral polio vaccine (OPV) to inactivated polio vaccine (IPV) through a phased approach, with the first step being the unprecedented introduction of IPV in the routine immunization (RI) program of 126 countries by the end of 2015 [2–5].

While the burden of rolling out IPV rested primarily on countries, the Global Polio Eradication Initiative established a global Immunization Systems Management Group (IMG) to manage and coordinate partners’ activities toward the attainment of this ambitious target. In particular, the IMG implementation subgroup was created to ensure that all OPV-using countries had access to the information, technical support, and financial resources needed to enable the introduction of at least 1 dose of IPV and the switch from trivalent OPV to bivalent OPV [6]. The IMG used a so-called tiering system to prioritize communication, technical assistance, and advocacy efforts towards countries with the highest polio risk (ie, tier 1 and tier 2 countries). In response to increasing global IPV supply constraints, the IMG prioritized supply to tier 1 and tier 2 countries and helped with the global coordination of these introductions [7, 8].

This article distills key lessons learned from the introduction process of IPV in 3 tier 1 polio countries (Cameroon, Kenya, and Nigeria), with the aim of informing future introductions of new vaccines (hereafter, “new vaccine introductions” [NVIs]). This should be valuable to the immunization community because the frequency of NVIs has increased and will continue [9, 10].

The article draws from the experiences of those actively involved in IPV introduction and a thorough review of key communications and documents from global and national task forces. Clinton Health Access Initiative (CHAI) staff in Cameroon, Kenya, and Nigeria worked on a day-to-day basis at the service of their governments to support the introductions, while other CHAI staff worked at the global level and participated in the IMG implementation subgroup. Thus, this article is informed by CHAI’s first-hand operational experiences, as well as CHAI’s extensive NVI experience [11].

The article is structured according to 6 key steps to introduce a new vaccine: (1) decision-making, (2) planning and project managing the introduction, (3) preparing the supply and cold chain, (4) financing the introduction, (5) launching the vaccine and postintroduction tracking, and (6) integrating the NVI within RI activities. It concludes by reflecting on the global coordination role of technical working groups, such as the IMG.

## DECISION-MAKING

The decision to introduce a vaccine into the national immunization schedule is made by the government, typically after considering the expected health impact, cost, and financing, as well as other health system issues [12]. Using those decision criteria, the introduction of IPV could have been unattractive for national expanded programs on immunization because use of IPV is primarily a risk mitigation strategy to prevent reemergence of polioviruses after eradication. This was especially a concern because of competing national priorities, including other NVIs, that would have averted more deaths. However, all 3 countries prioritized IPV introduction: it took national interagency coordinating committees only 2 months, in Kenya, 4 months, in Nigeria, and 5 months, in Cameroon, to officially decide to introduce IPV following the decision by Gavi (also known as the Global Alliance for Vaccines and Immunization) in November 2013 to support IPV introduction [13, 14, 15, 16].

There were 2 key enablers to these countries’ rapid decision to introduce IPV, both of which resulted from global players’ actions. First, Gavi eased its conditions for accessing financial support by lifting the requirement for cofinancing and the need to have >70% coverage by the third dose of a vaccine containing diphtheria-tetanus-pertussis (DPT3) [17]. This incentivized national immunization programs to align with the global polio eradication agenda and enabled countries to rapidly secure the financial support needed for IPV introduction. Second, the IMG allayed a significant concern expressed initially by key stakeholders in all case countries that mothers may not accept IPV because it would be the third injection at 14 weeks. IMG members had anticipated this issue [18] and shared evidence from countries such as Albania, which introduced IPV in similar conditions, that caregivers were ready to accept multiple injections, provided that the healthcare workers (HCWs) clearly communicated the benefits of these injections [19, 20].

## PLANNING AND PROJECT MANAGING THE INTRODUCTION

National NVI technical working groups (TWGs) are critical in building momentum for vaccine introduction, as well as in planning and managing its implementation. In Kenya, the national expanded program on immunization (EPI) and partners formed an IPV TWG in March 2015, along with subcommittees in training, social mobilization, and logistics. Weekly meetings helped keep the momentum necessary for the smooth preparation of IPV introduction. Similarly, in Nigeria the existing NVI subcommittee in the routine immunization working group supported IPV introduction. Cameroon, contrastingly, began the introduction process without a formal coordination body. However, as the country realized that critical activities such as document development were stagnating, the EPI decided to create an NVI TWG to oversee all preparatory activities. Terms of reference provided this new structure with a clear mandate, giving crucial accountability for introduction.

Countries must implement robust project management systems to ensure that a vaccine introduction is executed on time, on budget, and to the right quality. In Kenya, the first task of the TWG was to create a detailed project schedule in the form of a Gantt chart informed by lessons learned from previous NVIs. The Gantt chart front-loaded activities that were the primary causes of delays in previous introductions (eg, printing of training materials, delivery of advocacy materials, and receipt of vaccine clearance at port). Each TWG meeting began with a review of the previous meeting’s action steps and a careful review of progress with respect to the Gantt chart. This tool became increasingly important in the face of unplanned events, such as the IPV supply delays, which shifted the launch from June to October 2015. Updating the Gantt chart for all dependent activities once this constraint was known ensured that additional delays were minimized.

Finally, strong coordination with subnational stakeholders is paramount for a successful NVI because national rollout requires coordinating thousands of stakeholders at various levels and locations. Innovative telecommunication tools and strategic stakeholder engagements can be harnessed to improve the effectiveness of NVI processes. In Cameroon, for instance, the NVI TWG established weekly teleconferences with all regions, which enabled it to sensitize regional stakeholders and track the implementation of planned activities. This was possible because the TWG created detailed work plans and prelaunch checklists for each region. In Kenya, the TWG engaged subnational bodies by leveraging available forums, such as the EPI county annual work planning meetings, to cascade key information surrounding IPV rationale, schedules, and activities. They then used a WhatsApp group, which enabled EPI officers at all levels to share key best practices and track the delivery of the vaccines and materials on a regular basis.

## PREPARING THE SUPPLY AND COLD CHAIN

Global IPV shortages caused many countries to defer their introduction of IPV. While better measures are needed to minimize global supply issues, such issues cannot always be avoided during a NVI, so it is important that this risk be well communicated and managed across stakeholders. In the context of increasing global IPV supply constraints, the IMG’s prioritization of supply to tier 1 and tier 2 countries was a rational strategy to mitigate the risk of circulating vaccine-derived poliovirus and was transparently communicated to countries. Although even tier 1 priority countries encountered delays due to the shortages—Kenya and Nigeria had to delay their IPV introductions by 2 and 4 months, respectively—these were much shorter than those of most lower-tier countries [21, 22].

Kenya and Nigeria were notified of delays in receipt of their IPV supplies 2 and 3 months, respectively, before their planned launches. While this is a noticeable improvement in communication as compared to previous NVIs (notably rotavirus vaccine and pneumococcal vaccine), in which global supply delays were announced to some countries as late as 1 month before launch, these communications could come even earlier by alerting countries about key risks of supply delays even before they are confirmed.

Giving better information to countries about potential risks of supply delays would be valuable for them to build better contingency plans and minimize the impact on national immunization programs. It would also foster greater trust between global and local stakeholders. Within the country, global vaccine supply shortages can not only delay launch but also can cause the EPI to incur extra costs and hurt other program activities planned during that period. For example, the Kenya EPI team had planned to use the routine quarterly distribution systems from the national to regional levels to transport IPV in October 2015. However, because of the IPV shipment delay from July 2015 to November 2015, the EPI team had to spend extra funds for emergency transportation from national to regional stores. Several subcounties then waited for the next routine vaccines pickup in January to collect their IPV stock, thus further delaying IPV introduction in those areas, owing to a lack of funds for additional pickups.

Moreover, experience from IPV introduction highlights the need for strong multiyear cold chain planning and management, including activities for repair and maintenance systems. In Nigeria, a multiyear cold chain expansion plan that was developed in anticipation of pneumococcal and rotavirus vaccine introduction was easily updated to ensure adequate storage for IPV, as well. Such long-term cold chain planning is important to mitigate the significant lead time between identifying cold chain gaps and filling them on-site.

Conversely, weak cold chain systems present a significant risk to NVIs. In Cameroon, 1 month before IPV introduction, an assessment of the cold chain performance by use of temperature-monitoring devices showed that vaccines at the national store were being exposed to prolonged excursions. When EPI followed up on vaccine quality, they discovered that 447 200 IPV doses had been damaged (unpublished presentation, Agence Medicale Preventive-Gavi mission, July 2015). If cold chain capacity and functionality had been evaluated at least 10 months before the launch, EPI would have had the opportunity to implement necessary improvements before the introduction. On the positive side, Cameroon’s IPV damage served as an alarm bell regarding the cold chain system. Camaroon’s Ministry of Health now considers obtaining better cold chain equipment a priority, and Cameroon prioritized funds in its May 2015 Gavi Health System Strengthening application (53% of total funds) to enhance its cold chain.

The cold chain gaps in Cameroon also highlight the value of having a forum such as the IMG for relevant parties to discuss key issues that require country and global coordination. Initially, the Cameroon government was expected to pay for the damaged doses before delivery of the second shipment. This could have led to prolonged stock outs, given the limited liquidity of government funds. Through successful discussions among EPI, Gavi, and key partners from the IMG, a joint solution was found for the second shipment to be issued before reimbursement.

## FINANCING THE INTRODUCTION

Although Gavi-eligible countries received full financial support from Gavi for IPV procurement, operational costs (eg, for cold chain volume and HCW time) for delivering IPV are borne by public systems. For the IPV introductions in Cameroon, Kenya, and Nigeria, service delivery costs were not explicitly accounted for in government financial information because of the assumption that the costs were marginal and could be absorbed by the existing system. This lack of systematic review presents a risk in the long term on the quality of service delivery at the facility level that would need greater attention. Indeed, the number of HCWs might not increase sufficiently to cope with the growing number of vaccines being administered. In a 2012 survey in 6 countries, 61% of health facility respondents reported that workload had increased at the time of or just after a NVI [23]. Moreover, there is a shortage of HCWs in low-income and lower-middle-income countries, which highlights the need for substantial increases in full-time–equivalent staff for vaccination, owing to the current pace of NVIs [24].

Financing for introduction activities was one of the main needs in-country. The IMG played a key role in providing visibility on Gavi’s timelines for the release of vaccine introduction grant (VIG) funds. For instance, because the IMG was able to provide transparency about a 2-month delay in the release of the VIG to Nigeria, Nigeria was able to obtain prefunding arrangements with other partners, which enabled 2 states to introduce IPV in February 2015, before release of VIG funds. However, financing processes still compromised the timeliness of launches owing to delays at the country level in releasing funds to service delivery entities. Experiences during the IPV introductions highlight a need to smoothen in-country VIG release processes or to better factor long timelines into the choice of launch date. In Kenya, the VIG was delayed by 2 months owing to transfer challenges from UNICEF to the Ministry of Health because of lengthy fund disbursal procedures. This delay slowed the printing of important tools, such as the vaccine ledger books and the permanent register. This ultimately affected the quality of coverage estimates for the initial months of IPV delivery. This experience also highlights the need for ministries of health to budget introduction-associated funds so that, as much as possible, the activities that need to happen early (eg, printing and national training) do not depend on the VIG release date.

Moreover, communication of VIG rules should be enhanced. In Cameroon, the EPI team was not aware of the Gavi rule that all contracts greater than $10 000 need to be preceded by a tender. This was problematic because EPI intended to subcontract all IPV-related printing to the national printing house. After long discussions, it was possible for Cameroon to send the documents to this printing house, but it delayed the introduction process by 4 weeks.

## LAUNCHING THE VACCINE AND POSTINTRODUCTION TRACKING

NVI typically involves an official launch event to advertise and celebrate the availability of the new vaccine, but these events, which often involve busy, high-profile officials, should not delay the effective vaccine rollout. Owing to difficulties in scheduling the official launch event in Kenya and Nigeria, regions were requested to begin administering IPV beforehand. Kenyan HCWs were informed during training that they should start vaccination as soon as the vaccine was delivered.

**Figure 1.**
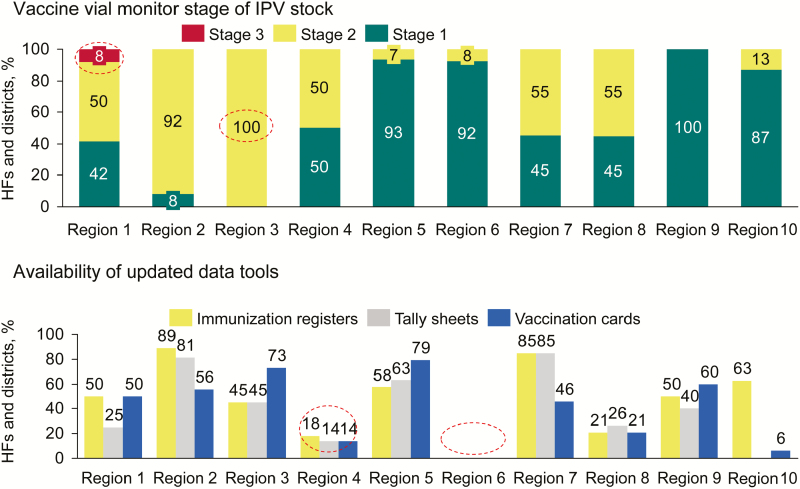
Issues with stock quality and availability of updated data tools among 239 health facilities (HFs) and 167 districts revealed by a postlaunch assessment conducted in September 2015, 2 months after vaccine introduction. Abbreviation: IPV, inactivated polio vaccine.

**Figure 2.**
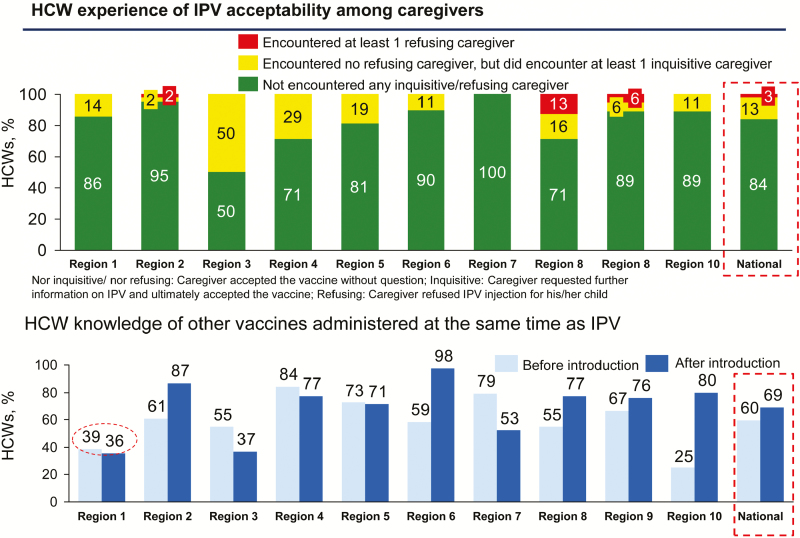
Postlaunch assessment conducted among 239 health facilities (HFs) and 167 districts in September 2015, 2 months after vaccine introduction, showed that, among 239 healthcare workers (HCWs), experience of inactivated polio vaccine (IPV) acceptability among caregivers was mostly good and HCW knowledge of all antigens given with IPV 1 week before and 8 weeks after introduction had generally improved.

Early postlaunch assessments allowed all 3 countries to quickly identify and mitigate bottlenecks to IPV uptake, accelerating an increase in coverage. In Cameroon, the EPI led telephone surveys 1 week before and 2 months after the launch, reaching approximately 90% of districts and 16% of health facilities. Figures 1 and 2 present some of the key results collected through the postlaunch assessments, highlighting specific gaps in updated data tool availability and stock quality (which could be traced back to the cold chain issue at the national store). Findings from these assessments enabled the EPI to take rapid corrective action, including printing and distributing additional data tools to enhance staff capacity and supplying IPV stock to specific regions where stock-outs were being experienced, such as the East region.

In Nigeria, the EPI scaled up state operation rooms to conduct regular IPV spot checks, monitor coverage, and coordinate corrective activities. For example, in Jigawa State, 35% of children receiving DTP3 were reported not to have received IPV between April and September 2015, which led the state to perform missed-opportunity tracking, on-the-job training for HCWs and data quality assessments. The coverage gap between DTP3 and IPV narrowed by 54% between October 2015 and April 2016.

One area of great importance for a smooth NVI is to closely manage stock availability and the implementation of eligibility policies at the subnational and facility levels. The global shortage led to more-constrained IPV stock allocation to all 3 countries. As a result, these countries temporarily adopted more-restrictive eligibility rules (eg, administration of IPV only at the 14-week visit, not to all children aged <1 year children), based on the experience from other NVIs that saw high demand from the so-called backlog cohort [25]. However, these restrictive policies were not consistently implemented by HCWs, resulting in IPV consumption exceeding supply and leading to IPV stock outs, as seen in Nigeria between March 2015 and June 2016 in all 36 states.

In addition to accelerating the uptake of a new vaccine, postintroduction assessments are useful to identify issues that can be avoided for future NVIs. For example, in Cameroon, an assessment found that the cascade-training model led to dilution of key messages to HCWs. As a result, the multidose-vial policy for IPV went largely unimplemented at first, and trainers during the April 2016 Switch preparations were asked to re-emphasize this policy. To ensure better knowledge transmission, Cameroon is considering cluster training for future NVIs.

As of June 2016, there is still a coverage gap between DTP3 and IPV in all 3 case countries (17 percentage points in Kenya [unpublished administrative data District Health Information System, District Vaccines Data Management Tool, June 2016], 9 percentage points in Cameroon [unpublished administrative data, WHO, June 2016], and 15 percentage points in Nigeria [unpublished administrative data, District Vaccines Data Management Tool April 2016). This shows that, even 6 months after launch, postintroduction tracking should continue because there are still steps to be taken to fully integrate the vaccine into the RI system.

## INTEGRATING THE NVI INTO RI

Countries should monitor and mitigate any negative impact of a new vaccine on the existing health system and, where possible, leverage the NVI as an opportunity to strengthen the immunization program and, more broadly, the health system. As described above, the introduction of IPV in all 3 countries has put pressure on the vaccine delivery system, such as cold chain capacity and HCW time. But DTP3 coverage rates in Kenya stayed constant, and in Cameroon and Nigeria they increased by 3 and 24 percentage points, respectively, from the month before the introduction to 6 months after, which showed that any negative IPV impact on RI was limited. Most HCWs thought that IPV did not hurt routine immunization—PCV and IPV postintroduction evaluations in Nigeria revealed that HCWs in 64% of states felt that IPV introduction had improved the vaccine program. For instance, in the north, HCWs felt the introduction helped to improve community-level social mobilization and awareness of immunization.

Key capabilities and experiences acquired for IPV introduction are useful for the other goals of the immunization programs. In Nigeria, the state operation rooms set up for IPV introduction are being used for other NVIs and to support RI activities; the experience with IPV also established regional officers’ ability to use data for action. For example, the operation room in Edo State identified prolonged power outages in cold stores of several local government areas. This report empowered the state team to conduct advocacy visits that resulted in the securing of funding from the local government authority chairmen for the reconnection of the cold stores to the power grid.

Good program management practices for NVIs can also help strengthen other EPI goals. In Cameroon, the NVI task team served as a model within EPI to build other working groups, such as the Cold Chain Logistics Group, because of its success in coordinating all stakeholders towards a common agenda.

## CONCLUSIONS

On the basis of experience with IPV introduction in Cameroon, Kenya, and Nigeria, we identified some key lessons learned that can benefit future new vaccine introductions. These IPV introductions highlight 3 critical components for NVI success in the planning and project management stage: (1) the existence of a national NVI technical working group, (2) robust project management systems for accountability, and (3) strong communication and coordination with subnational stakeholders.

**Table 1.** Key Best Practices for New Vaccine Introductions (NVIs) Identified During Inactivated Polio Vaccine Introduction

Key Step for NVI	Global	National	Best Practice Identified
**Decision-making**	X		Global policies on cofinancing and conditionality are effective tools to incentivize countries to introduce vaccines in alignment with global targets
	X		Sharing evidence globally from early NVI–adopting countries can reduce other countries’ concerns and facilitate NVI decision-making
**Planning and project management of the introduction**		X	Weekly NVI TWGs with clear terms of reference are critical in providing oversight, accountability, and momentum for NVI
		X	Robust project management systems should plan for front-loading of critical activities and include a weekly review of progress against project schedule
		X	Strong coordination with subnational stakeholders can leverage innovative means of communication, such as WhatsApp and weekly teleconferences
**Preparing the supply and cold chain**	X		When faced with global antigen shortages, tiering of countries on the basis of risk allows for transparent, rational supply prioritization
	X		Notifying countries of likely supply delays before they are confirmed enables EPIs to build stronger contingency plans, minimizing costs and further delays
		X	Prioritizing multiyear cold chain planning and building robust repair and maintenance systems averts the risk of vaccine damage
	X		Partner coordination forums that link global and country levels are useful, to agree on mitigation activities when crises arise
**Financing the introduction**		X	The marginal impact of each NVI on operational costs (eg, HCW time and cold chain) needs to be carefully analyzed to avoid compromising the quality of service delivery
		X	MoHs should budget introduction funds such that the activities that need to happen early (eg, printing and national training) are not dependent on VIG release
	X		Transparency on VIG delays allows countries to take mitigating action, such as seeking prefinancing from other partners
		X	Countries need to smoothen the often lengthy in-country VIG release process or set a realistic launch date that factors usual national disbursement timelines
	X		Communication of global partners’ policies should be enhanced so that EPIs are aware of all requirements related to fund use
**Launching the vaccine and postintroduction tracking**		X	NVI administration should not be held up because of official ceremonies
		X	Early postlaunch assessments (<2 months after vaccine rollout) allow EPIs to quickly identify and mitigate bottlenecks to vaccine uptake
		X	Subnational operation rooms can allow each region to conduct regular spot-checks and coordinate corrective activities
		X	Close monitoring of stock levels and enforcement of eligibility policies in-country ensure that the vaccine is available for the target cohort
		X	Postintroduction tracking needs to continue until target coverage is reached, to ensure that the NVI is fully integrated into the RI system
**Integrating the NVI within RI system**		X	Countries should monitor the existing health system to ensure that there is no negative impact from NVIs and that opportunities for improvements through NVI activities are leveraged
		X	EPI teams should adapt good NVI program management practices (eg, creation of operation rooms) to accomplish other immunization goals

Abbreviations: EPI, expanded program on immunization; HCW, healthcare worker; MoH, ministry of health; RI, routine immunization; TWG, technical working group; VIG, vaccine introduction grant.

They show that in-country preparations for vaccine supply and cold chain readiness for introduction must be thorough or they could become severe bottlenecks to NVI. They indicate how often financing remains a barrier to a successful and timely launch, as country budgeting and VIG release processes still need to be improved. They also pinpoint the usefulness of systems and management processes (eg, postlaunch assessments and operation rooms) used after the launch to swiftly identify challenges and implement corrective actions. Last, the case studies show the ability to leverage NVIs to reinforce RI activities and, conversely, the importance of strong vaccine delivery systems for successful NVIs.

The introduction of IPV provides insight into the key roles that global working groups such as the IMG can play to bring about, facilitate, and support robust introduction of new vaccines nationwide. The IMG facilitated introductions in-country. It provided a useful communication channel for updates on vaccine supply and VIG dispersal, Gavi policies, and best practices. Because of the political clout of its members, it helped achieve faster resolution between global and in-country partners when major issues took place, such as the IPV damage in Cameroon.

The IPV experiences also allow us to assess how such global support for vaccine introductions can be further enhanced. Given that an IMG-type structure relies on the strength of its member organizations to relay critical updates to the field, membership could be further expanded to include more medium-sized implementation partners. In addition, having greater representation by ministries of health themselves would enable the IMG to better take into account the preferences of countries, for instance, regarding when and how to communicate supply delays. In the case of IPV introduction, partners shared critical supply delay risks to countries only once they were confirmed, thus limiting countries’ ability to mitigate further introduction delays, costs, and disruption on other immunization goals. Last, an IMG-type structure could be complemented with a peer-to-peer structure for EPI teams to share best practices, tools, and training materials regionally or globally, given the degree of similarity in issues experienced across countries.

## Notes

***Acknowledgments.*** We thank personnel of the ministries of health in Cameroon, Kenya, and Nigeria and members of the Immunization Systems Management Group, for their commitment to the Routine Immunization program and their efforts in the successful introduction of IPV.

***Financial support.*** This work was supported by the Bill and Melinda Gates Foundation (grant OPP1084393).

***Supplement sponsorship.*** This work is part of a supplement coordinated by the Task Force for Global Health with funding provided by The Bill and Melinda Gates Foundation and the Centers for Disease Control and Prevention.

***Potential conflicts of interest.*** All authors: No reported conflicts. All authors have submitted the ICMJE Form for Disclosure of Potential Conflicts of Interest. Conflicts that the editors consider relevant to the content of the manuscript have been disclosed.
